# Extramedullary tibial guide orientation in TKA personalized alignment: validation of a trigonometric method

**DOI:** 10.1007/s00402-025-06099-x

**Published:** 2025-11-04

**Authors:** Rosario Junior Sagliocco, Filippo Leggieri, Andrea Baldini, Domenico Andrea Campanacci, Roberto Civinini, Matteo Innocenti

**Affiliations:** 1 Scuola di Specializzazione in Ortopedia e Traumatologia, Dipartimento di Scienze della Salute, Università degli Studi di Firenze, Florence, Italy; 2 U.O.C. Ortopedia e Traumatologia, Presidio Ospedaliero dei Pellegrini, Naples, Italy; 3 Istituto Fiorentino di Cura e Assistenza (IFCA), Florence, Italy

**Keywords:** Total knee arthroplasty, Personalized alignment, Tibia resection, Kinematic alignment, Extrameduallry guide

## Abstract

**Introduction:**

Achieving personalized alignment in total knee arthroplasty (TKA) with conventional instrumentation remains challenging. This study validates a novel trigonometric formula that calculates the exact lateralization needed for the tibial extramedullary guide based on preoperative weight-bearing X-rays.

**Methods:**

We retrospectively analysed 196 patients who underwent TKA between November 2018 and June 2023. Inclusion criteria: patients with preoperative weight-bearing AP lower limb X-rays aged 18 or older. Exclusion criteria: previous total hip arthroplasty, those without consent. The formula LAT = LENG(S) × sin α angle calculated tibial guide lateralization, where LAT was the lateralization distance, LENG(S) was the tibial length from radiographs, and α angle was the tibial coronal correction angle. The true radiographic lateralization was measured to validate the formula’s accuracy and defined a “safety zone” representing acceptable surgical margins to validate the formula’s accuracy and defined a “safety zone” representing acceptable surgical margins. The Intraclass Correlation Coefficient (ICC) was used to test for the measurement consistency among observers. 95% Clopper-Pearson Confidence Interval was calculated for the frequency of lateralization falling within a “safety zone”. A T-test compared LAT measurements with true radiographic lateralization.

**Results:**

ICC showed that 97.2% of lateralization measurements fell within the defined “safety cone” (95% CI 93.9–98.9%). Inter-observer reliability was high (ICC 0.91). No differences were found between the formula-derived measurements and the true radiographic lateralization. The 95% Clopper-Pearson Confidence Interval was 93.9–98.9%. LAT was found to fall outside the safety cone with a total mean of 2.3° (range 1–5) in 2.8% of the cases, with a mean error in the degree of proximal tibial cut of -0.67° (range − 1 - +1). No association between CPAK and cases within or outside the safety cone was found (χ²= 5.014, *p* = 0.658).

**Conclusions:**

This validated trigonometric formula enables surgeons to accurately calculate tibial guide lateralization for personalized alignment using only conventional instrumentation and standard radiographs. The method’s 97.2% accuracy within safe surgical margins supports its use as a reliable preoperative planning tool for personalized TKA alignment without requiring specialized software or robotic assistance.

## Introduction

Personalized alignment in total knee arthroplasty (TKA) represents a paradigm shift from traditional mechanical alignment [1].

The application of personalized alignments varies in difficulty by the desired alignment philosophy. Mechanical alignment using standard extramedullary instrumentation is straightforward, producing neutral cuts perpendicular to the mechanical axis. Unrestricted kinematic alignment (uKA) is similarly simple, requiring cuts parallel to existing surfaces [2]. However, restricted kinematic alignment (rKA) presents significant technical challenges, demanding precise partial deformity corrections with specific resection angles that neither match the mechanical axis nor replicate the original deformity [3]. While distal angled cutting bushings for the femoral side are available in most instrumentation sets and allow for easier reproduction of the planned resection angle, this challenge is especially pronounced on the tibial side, where accurate lateralization of the extramedullary guide becomes critical to achieve the desired resection angle [4, 5].

Without robotic assistance or patient-specific instrumentation, surgeons must rely on conventional instrumentation to create these “fine-tuned” cuts, while the intramedullary tibial guidance systems are inherently constrained by perpendicularity. The absence of a reliable, quantifiable method for extramedullary guide positioning represents a substantial barrier to the wider adoption of personalized rKA techniques using conventional instrumentation. Therefore, developing a precise, reproducible method for extramedullary tibial guide positioning is essential to facilitate accurate implementation of personalized alignment principles in routine clinical practice, with conventional instrumentation.

The aim of this study was to introduce a novel trigonometric method for preoperative planning of the exact amount of lateral shift to be applied to the tibial extramedullary guide on the intermalleolar axis. This method employs only weight-bearing lower limb X-rays, making it accessible and cost-effective for routine clinical practice while maintaining the precision necessary for successful rKA implementation.

## Materials and methods

### Population and design

This study presents a single-cohort retrospective analysis of prospectively collected data from 196 randomly selected patients. The cohort was drawn from an internal electronic database of patients who underwent TKA surgery at a single institution between November 2018 and June 2023.

Inclusion criteria comprised patients aged 18 or older who had preoperative weight-bearing AP lower limb X-rays before TKA surgery. Participants with previous total hip arthroplasty surgery, or those whose consent was not obtained were excluded.

These criteria were selected to ensure methodological validity while maintaining ethical standards. Weight-bearing AP lower limb X-rays were essential as they provide visualization of alignment under load, critical for personalized knee planning. By including only patients who had already undergone TKA surgery with existing radiographs, we avoided exposing additional patients to unnecessary radiation. We excluded patients with previous total hip arthroplasty to eliminate potential confounding variables that might alter natural lower limb mechanical alignment. All inclusions adhered to Declaration of Helsinki principles with appropriate patient consent. The Local Ethics Committees reviewed the study protocol and determined that no ethical approval was required, given the purely retrospective and observational nature of the design.

### Outcome measures

#### Primary outcome

The primary outcome measure was the accuracy of the trigonometric formula (LAT), defined as the percentage of lateralization measurements falling within the defined “safety cone” when compared to true radiographic lateralization ($\:{LAT}_{true}$).

#### Secondary outcomes

To establish the clinical reliability of the trigonometric formula, we evaluated inter-observer reliability for radiographic measurements using the Intraclass Correlation Coefficient (ICC) among independent observers. Formula validation was performed by statistically comparing the formula-derived lateralization measurements (LAT) with the true radiographic lateralization ($\:{LAT}_{true}$) using an independent t-test. This comparison established whether the trigonometric calculation accurately predicted the radiographic reality of guide positioning. The dimensions of the “safety cone” were measured in millimeters to quantify the acceptable surgical margin for extramedullary guide lateralization. For cases where measurements fell outside the safety cone, a comprehensive error analysis was conducted. This included measuring the degree of deviation from the safety cone and calculating the resulting error in the proximal tibial cut angle. Additionally, key parameters such as mMPTA, LENG(S), safety cone dimensions, LAT, and $\:{LAT}_{true}$ were compared between cases that fell within versus outside the safety cone to identify any systematic patterns or predictive factors. Finally, we investigated whether specific CPAK phenotypes were associated with a higher likelihood of measurements falling within the safety cone.

### Radiographic data collection

Comprehensive radiographic measurements were collected for each patient using standardized weight-bearing anteroposterior (AP) lower limb X-rays with a squared grid calibration. Each square in the calibration grid measured 50 mm × 50 mm, providing a consistent reference for all measurements.

#### Angular measurements

The following angular parameters were evaluated:

- Mechanical Medial Proximal Tibial Angle (mMPTA): The angle formed between the mechanical axis of the tibia and the tibial plateau.
- Mechanical Lateral Distal Femoral Angle (mLDFA): The angle formed between the mechanical axis of the femur and the distal femoral joint line.
- Coronal Plane Alignment of the Knee (CPAK): Used to classify the phenotype of knee deformity [1].
- Tibial resection angle (α angle): The planned tibial resection angle according to restricted kinematic alignment (rKA) guidelines [3], representing the desired residual varus angle to be achieved postoperatively on the tibia. This angle determines the coronal plane orientation of the tibial cut.
- Tibial resection line: The postoperative coronal tibial joint line.

#### Tibial length measurement protocol: calibration square-based measurement of the tibial length- LENG(S)

**Fig. 1:**
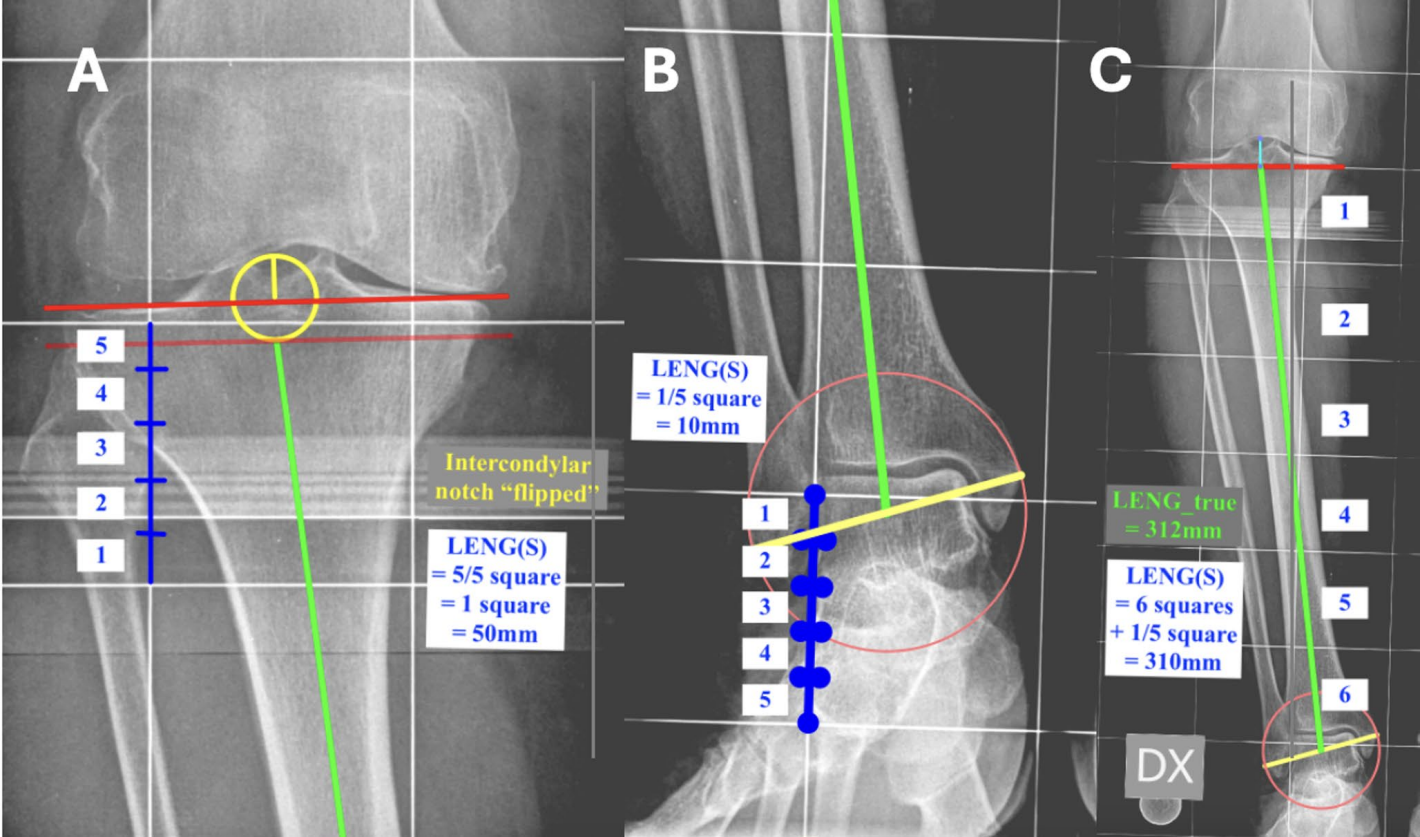
The image shows how tibial length was measured according to the two methods: direct millimetric measurement ($\:{LENG}_{true}$) and calibration square-based measurement (LENG(S)). **A** The image shows a detail of the proximal reference point for tibial length and a detail of the “fifth of a square” rule to measure LENG(S). The fifth is always rounded up to the nearest higher fifth. **B** The image shows a detail of the distal reference point for tibial length and a detail of the “fifth of a square” rule to measure LENG(S). The fifth is always rounded up to the nearest higher fifth. **C** The image shows how $\:{LENG}_{true}\:$and LENG(S) measure tibial length between the proximal and distal reference points

The calibration square-based measurement of the tibial length (LENG(S)) was measured between the proximal reference point and the distal reference point (Fig. 1).

The proximal reference point was set at the level where the intercondylar eminence inverts relative to the joint tangent (Fig. 1A). The intercondylar eminence inversion point was selected as the proximal reference for tibial length measurement based on anatomical and practical considerations [6–9]. Unlike the joint tangent, which varies with arthritic changes and can be difficult to define consistently in degenerative knees, the intercondylar spines remain relatively preserved across disease stages. This landmark provides superior radiographic visibility and inter-observer reliability compared to joint line measurements, particularly in cases with severe wear or osteophyte formation. This measurement approach also reflects the mechanical relationship between the extramedullary guide and tibial anatomy during surgery. Since the guide rests against the anterior tibial cortex rather than the joint surface, referencing measurements from a stable bony landmark creates a more direct correlation with intraoperative guide positioning. Additionally, this method maintains consistency across varying degrees of arthritis, ensuring formula accuracy in both early and end-stage disease.

The distal reference point was established at the midpoint of the intermalleolar axis at the talus (Fig. 1B) [10, 11]. LENG(S) was calculated by summing the complete squares and any partial squares (measured in fifths) (Fig. 1C). For partially intersected squares at either boundary, measurements were rounded up to the nearest fifth. The final number of squares and the “fifths of a square” is multiplied by the length of the grid square which is 50 mm (Fig. 1C). The result is the length of the tibia in millimetres measured by the calibration square-based measurement.

#### Tibial length measurement protocol: direct millimetric measurement of the tibial length-$\:{LENG}_{true}$

Using the same anatomical reference points as the square-based method, the direct millimetric measurement of the tibial length ($\:{LENG}_{true})$ was measured in millimetres along the tibial mechanical axis and was recorded as the true tibial length (Fig. 1C).

#### Intermalleolar axis measurement

To determine the intermalleolar axis on anteroposterior radiographs, a circle was drawn encompassing the distal tibiofibular region at the level of the ankle joint. The diameter of this circle was positioned to capture the most lateral point of the lateral malleolus and the most medial point of the medial malleolus. The intermalleolar axis was then defined as the line connecting these two extreme points, representing the maximum transverse dimension between the malleoli.

### The trigonometric formula (Fig. 2)

The proposed formula to measure the millimetres of lateralization that the extramedullary guide must impart intraoperatively is based on the trigonometric *sine* function of the tibial resection angle using the length of the tibia based on the calibration square-based measurement of the tibial length (LENG(S)). However, $\:{LENG}_{true}$ can also be used instead.

**Fig. 2:**
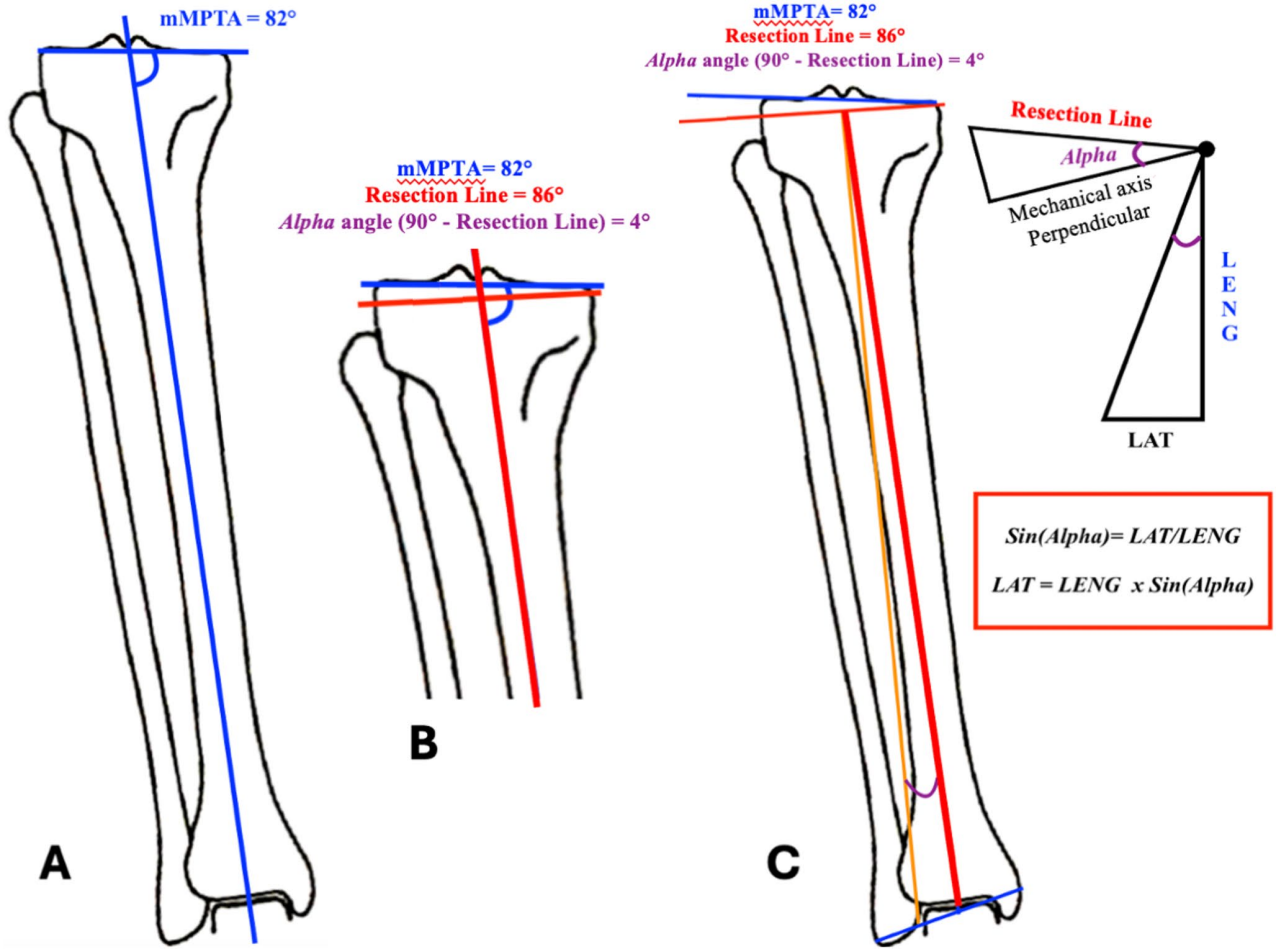
The image shows the rationale of the trigonometric formula. (A) mMPTA is calculated to assess the patient’s coronal tibial deformity. (B) From the mMPTA and following the rKA principles, *α* angle is chosen. This angle represents the desired postoperative coronal tibial deformity angle. (C) The lateralization of the extramedullary guide is measured with the *sine* function of the *α* angle. Tibial length can be measured as LENG(S) or $\:{LENG}_{true}$ in the clinical scenario, depending on the surgeon’s preference

*Step* 1 – Deformity analysis: Detect mLDFA and mMPTA (Fig. 2A).

*Step* 2 – Deformity classification: Classify the phenotype in the coronal plane according to the CPAK classification [1].

*Step* 3 – Tibial resection correction: Establish the amount of coronal adjustment that must be made for the tibial resection according to the rKA algorithm to correct the deformity [3]. The angle of correction to be imparted with the tibial resection is defined as $\:a$ angle (Fig. 2B).

*Step* 4 – Application of the trigonometric formula: Apply the following formula on the X-ray (Fig. 2C):

1 $$\:LAT=LENG\left(S\right)\times\:\:sin\:a\:$$

where *LAT* represents the lateralization in millimeters of the extramedullary tibial guide, measured from the center of the intermalleolar axis. As shown by Eq. (1), multiplying the value of $\:LENG\:\left(S\right)$ by the *sine* of *α* angle *(sin α*) allows for the calculation of the lateral shift in millimeters of the guide along the intermalleolar axis from its midpoint (Table 1).

**Table 1:** Millimetres of tibial guide lateralization for different tibial lengths [LENG(S)] and tibial varus correction angles

LENG(S) mm	Sin 1° (0.0175)	Sin 2° (0.035)	Sin 3° (0.052)	Sin 4° (0.070)	Sin 5° (0.087)
260	4.55	9.1	13.52	18.2	22.62
270	4.725	9.45	14.04	18.9	23.49
280	4.9	9.8	14.56	19.6	24.36
290	5.075	10.15	15.08	20.3	25.23
300	5.25	10.5	15.6	21	26.1
310	5.425	10.85	16.12	21.7	26.97
320	5.6	11.2	16.64	22.4	27.84
330	5.775	11.55	17.16	23.1	28.71
340	5.95	11.9	17.68	23.8	29.58
350	6.125	12.25	18.2	24.5	30.45
360	6.3	12.6	18.72	25.2	31.32
370	6.475	12.95	19.24	25.9	32.19
380	6.65	13.3	19.76	26.6	33.06
390	6.825	13.65	20.28	27.3	33.93
400	7	14	20.8	28	34.8
410	7.175	14.35	21.32	28.7	35.67
420	7.35	14.7	21.84	29.4	36.54
430	7.525	15.05	22.36	30.1	37.41
440	7.7	15.4	22.88	30.8	38.28
450	7.875	15.75	23.4	31.5	39.15

### Radiographic validation of the formula

To validate the method, the true radiographic lateralization ($\:{LAT}_{true})$ from the intermalleolar axis center was measured with TraumaCad digital software. The following steps were followed to obtain $\:{LAT}_{true}.$

**Fig. 3:**
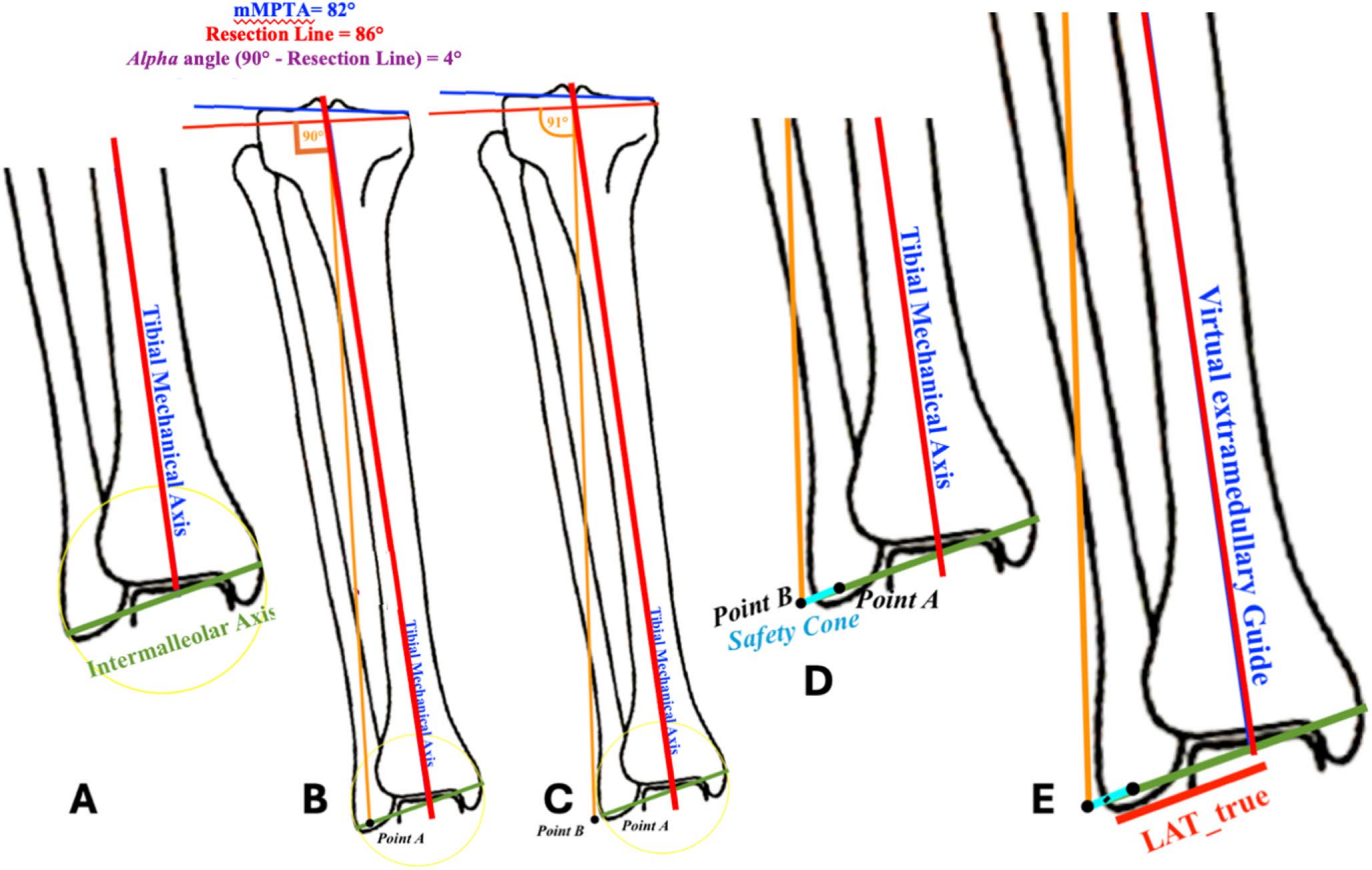
The image shows how the trigonometric formula was validated. The lateralization of the extramedullary guide was validated by measuring the same distance with two different methods: LAT using the trigonometric formula and $\:{LAT}_{true}\:$measuring the lateralization of the mechanical axis/virtual extramedullary guide along the intermalleolar axis. **A** When the tibial mechanical axis is measured, it falls in the midpoint of the intermalleolar axis. **B** The mechanical axis is shifted laterally until the angle between the mechanical axis and the resection line is equal to 90°. The point measured as the intersection between the intermalleolar axis and the tibial mechanical axis perpendicular to the resection line is Point (**A**, **C**) A further shift is imparted laterally to the mechanical axis. The point measured as the intersection between the intermalleolar axis and the tibial mechanical axis when the angle between the mechanical axis and the resection line is 91° is named Point (**B**, **D**) The line between Point A and Point B is named Safety Cone, and this is the width where the tibial mechanical axis remains perpendicular to the resection line. **E** The distance between the midpoint of the intermalleolar axis and the midpoint of the safety cone is the true radiographic lateralization of the extramedullary tibial guide ($\:{LAT}_{true}$)

1. The same steps from 1 to 3 from the previous paragraph (2.4 The trigonometric formula) were performed to find the desired *α* angle according to the rKA principles (Fig. 3A, B).
2. The midpoint of the intermalleolar axis was calculated in the X-Ray (Fig. 3A).
3. The mechanical axis of the tibia was then lateralized until it reached a position perpendicular to the planned proximal tibial resection line according to the rKA principles (*α* angle). In that position the mechanical axis was perpendicular to the kinematically aligned tibial joint line measuring an angle of 90° (Point A) (Fig. 3B).
4. From Point A, the mechanical axis of the tibia was further lateralized along the intermalleolar axis until it was at 91° (Point B) with respect to the kinematically aligned tibial joint line (*α* angle) (Fig. 3C).
5. The distance between Point A and Point B represents the amount of lateralization that the intermalleolar axis can achieve while still being perpendicular to the kinematically aligned tibial joint line measuring an angle of 90°. The distance was defined as “safety cone” as it represents the lateralization that does not change the coronal deformity correction (Fig. 3D).
6. The true radiographic lateralization ($\:{LAT}_{true})$ is identified by the distance on the intermalleolar axis from its midpoint to the center of the safety cone (Fig. 3E). This distance defines the amount of true lateralization of the extramedullary guide that must be imparted to achieve the correct position to cut the tibial joint line to obtain the residual varus mMPTA. $\:{LAT}_{true}$ was measured to validate the accuracy of LAT which was the lateralization to impart on the extramedullary guide measured with the trigonometric formula.

### Risk of bias

To limit potential sources of bias, all procedures were standardized for each patient. Data collection was performed by two independent orthopedic surgeons and double-checked by a trained resident to assess for any discrepancies or data entry errors. Any discrepancy was settled by a fourth senior surgeon.

### Data analysis

Descriptive statistics, such as means and ranges, were calculated for continuous variables, while categorical variables were summarized using frequencies and percentages. The Intraclass Correlation Coefficient (ICC) was used to test for the measurement consistency among observers. The 95% Clopper-Pearson confidence interval was calculated for the proportion of *LAT* measurements falling within the safety cone, providing an estimate of the accuracy of this proportion. An independent *t-test* compared *LAT* measurements with true radiographic lateralization $\:{LAT}_{true}$. For cases outside the safety cone, a Mann-Whitney U test evaluated the differences in mMPTA, length of the tibia $\:LENG\:\left(S\right)$, safety cone measurements, *LAT*, and $\:{LAT}_{true}$. The *Chi-Square* (*χ²*) test assessed the association between CPAK classification and *LAT* measurements’ likelihood of falling within the safety cone. Statistical significance was set at *p* < 0.05. Statistical analysis and data collection were performed using SPSS v22 (IBM SPSS Statistics, Armonk, New York, USA).

## Results

The ICC showed a concordance rate of 0.91 (*95%CI* = 0.86–0.94) among observers for radiographic measurements. Knees were classified according to the CPAK [1] as follows: 82 type I (41.8%), 44 type II (22.4%), 16 type III (8.2%), 15 type IV (7.7%), 25 type V (12.8%), 12 type VI (6.1%),1 type VII (0.5%), 0 type VIII (0%), 1 Type IX (0.5%). The mean mLDFA measurements were 88° (range, 78–94) and the mean mMPTA measurements were 86° (range, 74–108). The mean “safety cone” measurement was 6.74 mm (range, 5.3–8.6 mm).

The lateralization measurements obtained using the proposed formula ($\:LAT)$ were found to fall within the safety cone for 97.2% of the cases. The 95% Clopper-Pearson confidence interval for the proportion of $\:LAT$ falling within the safety cone was 93.9–98.9%. In the 2.8% of the cases, $\:LAT$ was found to fall outside the safety cone with a total mean of 2.3° (range 1–5), with a mean error in the degree of proximal tibial cut of −0.67° (range −1 - +1). To further assess the 2.8% of cases outside the safety cone, Mann-Whitney U test was performed showing no differences for MPTA, $\:LENG\:\left(S\right)$, safety cone, $\:LAT$, and $\:{LAT}_{true}$ between cases within and outside the safety cone. No association between CPAK and cases within or outside the safety cone was found (*χ²*= 5.014, *p* = 0.658). No difference was found between $\:LAT$ and $\:{LAT}_{true}$ measurements (*t*(198) = 1,860, *p* = 0.64).

## Discussion

The main aim of this study was to validate a trigonometric formula that allows surgeons to determine the precise millimeters of lateralization required for the extramedullary tibial guide to achieve a personalized tibial resection angle. Through the proposed formula, surgeons can calculate the exact amount of lateral shift needed based on tibial length measurements - whether obtained in millimeters from calibrated radiographs, through digital planning software, or by counting calibrated squares on analog radiographs. This approach enables surgeons to achieve a customized degree of correction with planned residual varus, ultimately allowing for personalized tibial cuts without requiring advanced navigation, robotic systems, or custom-made instrumentation.

**Fig. 4:**
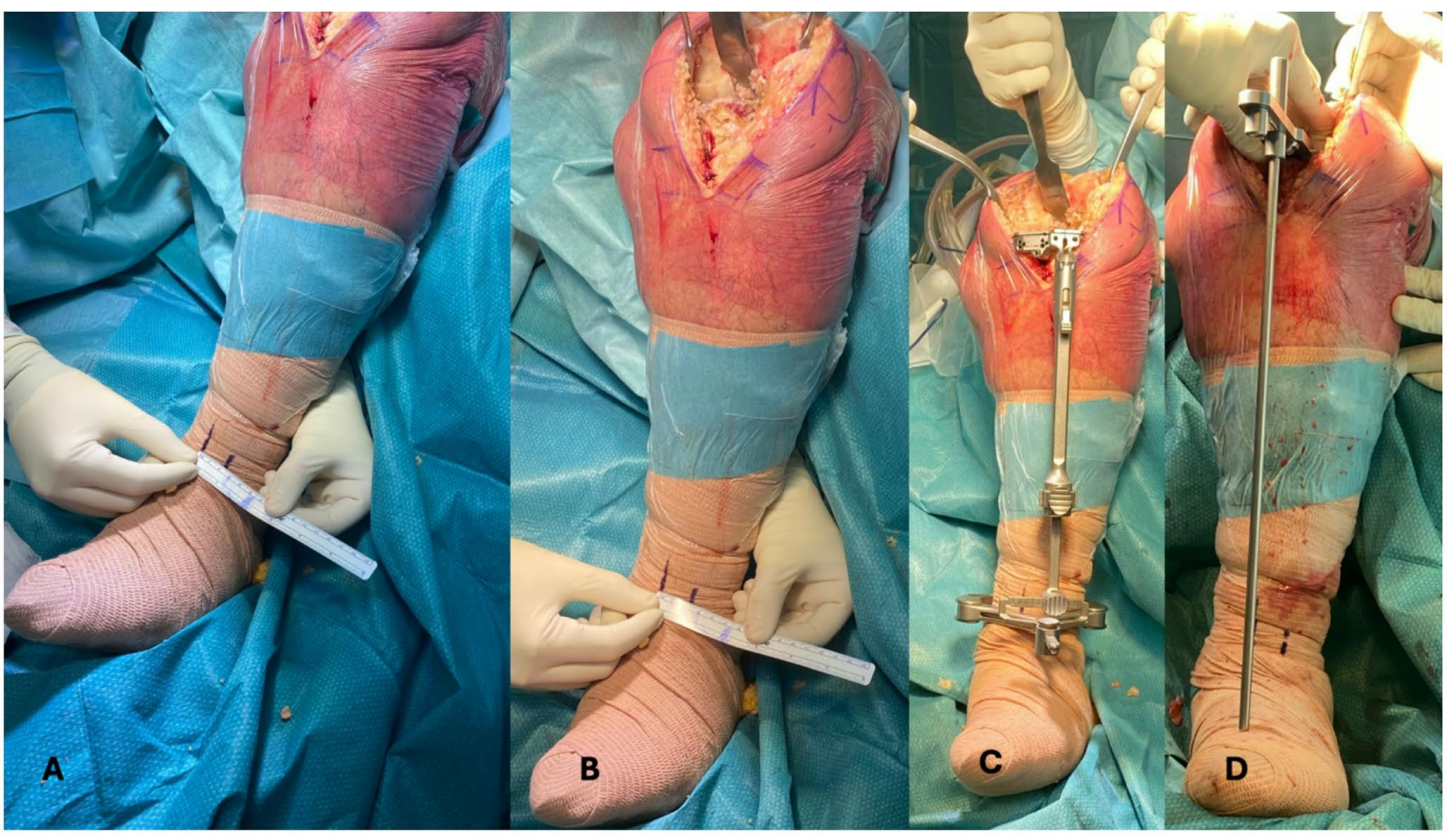
The image demonstrates the clinical application of the trigonometric formula for extramedullary tibial guide positioning in personalized knee alignment. **A** Measurement of the required lateralization distance (LAT) at the ankle level from the midpoint of the intermalleolar axis. **B** Close-up detail of the measurement process, showing how the exact lateralization distance is marked intraoperatively based on preoperative calculations. **C** Positioning of the tibial guide lateralized according to the trigonometric formula, allowing the guide to be placed at the exact calculated distance from the midpoint of the intermalleolar axis **D** Placement of 2° varus wedge on the tibial plateau to confirm the planned 2° varus angle cut. The extramedullary alignment guide confirms neutral mechanical alignment is achieved after accounting for the 2° wedge, validating that the precise 2° tibial varus cut has been accomplished

The clinical implementation of this method follows a straightforward intraoperative workflow (Fig. 4). During the preoperative planning phase, the surgeon calculates the lateralization distance (LAT) in millimeters using the trigonometric formula. Intraoperatively, the extramedullary guide is first positioned to establish a neutral tibial alignment, and this reference point is marked with a surgical pen on the patient’s ankle. Using a ruler, the surgeon then measures and marks a second point on the ankle, corresponding to the calculated lateralization distance. By repositioning the tibial guide from the first to the second mark, the surgeon achieves the desired degree of correction. This technique provides a reproducible and precise method for implementing personalized tibial resection angles using conventional instrumentation.

The main finding of this study was that 97.2% of the *LAT* measurements fell within the safety cone, with a 95% Clopper-Pearson confidence interval of 93.9–98.9%, demonstrating the formula’s relevance and potential efficacy in ensuring appropriate lateralization of the extramedullary tibial guide within safe surgical margins in the clinical scenario.

For the 2.8% of cases where LAT measurements fell outside the safety cone, further analyses revealed no differences in key parameters (mMPTA, $\:LENG\left(S\right)$, safety cone, *LAT*, and $\:{LAT}_{true})$ between these cases and those within the safety cone. This absence of association suggests that outliers are not attributable to systematic errors or identifiable patient characteristics based on the variables measured. The lack of differences between LAT and $\:{LAT}_{true}$ measurements further validate the formula’s accuracy, implying consistency between the formula-derived lateralization measurements and the true radiographic lateralization.

The study also demonstrated high inter-observer reliability for radiographic measurements, with an intraclass correlation coefficient (ICC) of 0.91, indicating consistent and reproducible measurement techniques across different observers.

Strategies commonly employed for tibial resection with conventional instrumentation involve positioning the vertical branch of the guide on the trajectory of an axis perpendicular to the planned resection viewed on X-ray, without having any clear reference. The lateral malleolus angle (LMA) might be used as a reference [12]; this is the angle between the tibial axis and the external surface of the lateral malleolus, corresponding to a value of approximately 5.5°± 0.5° By aligning the tibial guide with the axis that extends from the knee’s center to the outer surface of the lateral malleolus, a tibial varus cut of approximately 5.5°±0.5° can be achieved [12]. Other authors prefer to adjust the varus-valgus angle of tibial resection by placing an angel wing in the guide saw slot until it is parallel to the proximal tibial joint surface after compensating for wear [2]. Our trigonometric approach appears to offer accuracy, flexibility, and ease in determining the extent of lateralization.

One of the rationales behind this method was to optimize resource allocation while maintaining accuracy for TKA surgery. Conventional instrumentation in TKA has been demonstrated to offer cost-effectiveness without compromising clinical outcomes when compared to robotic-assisted surgery [13–15]. Long-term follow-up studies have shown equivalent 10-year survival rates between the two approaches [13–15]. Furthermore, some investigations have reported additional benefits with conventional methods, including reduced tourniquet time and shorter overall surgical duration [13–15].

The search for a trigonometric formula ensuring accurate tibial bone cuts was also driven by the necessity to establish a precise method for performing personalized alignments with conventional instrumentation. While the literature acknowledges the superiority of robotic systems in restoring original anatomy and coronal alignment, it demonstrates no significant clinical differences between the two methods [16]. The intentional application of the rKA algorithm was based on its ability to predict the angle of resection following specific guidelines and principles. This differs from other personalized alignment philosophies where the angularity of resection is deduced in at least one of the two bones based on ligament tensioning [17]. In the case of rKA, minor soft tissue releases are permitted in certain instances to achieve the correct balancing [3, 17, 18]. The focus on coronal plane analysis is supported by comparative studies between robotic and conventional surgery, which demonstrate no significant differences in tibial coronal and sagittal alignments [19].

Radiographic validation revealed a 1° less than planned in 2% of cases and a 1° more than planned in 1% of cases, resulting in an overall margin of error of ± 1° in only 3% of the cases. However, it is important to mention that the radiographs of those cases exhibited marked tibial malrotation, particularly affecting the overall measurement accuracy. Consequently, it is challenging to definitively determine whether the formula is more reliable than radiographic calculations of the lateralization in these cases. This limitation is inherent to the retrospective nature of the radiographic validation. The inclusion of such radiographs for the validation of the formula was intentional to address potential issues in tele-radiography with incorrect rotations. In such instances, we recommend adhering to the principles of rKA while aiming for a final aHKA of ± 2°. By narrowing the safety range, a potential error of 1° would remain within the limits prescribed by rKA. Another limitation was the inability to detect the extent of pre-arthritic tibial deformity in the presence of severe bone wear. In such scenarios, evaluation of pre-arthritic mMPTA may overestimate varus deformity, particularly when bone loss is medial. This challenge is not unique to our method but is common to any planning approach based on imaging studies conducted after the development of bone loss. In these cases, the deformity assessment on the contralateral healthy or less affected limb may prove beneficial, as reported in anecdotal cases [20].

For this study, we chose to utilize the CPAK classification for analyzing deformities; however, any classification system or methodology for studying coronal deformity can be used as the foundation for determining the desired degree of correction. Similarly, we employed the rKA philosophy, as it represents the standard clinical practice at our institution for non-robotic-assisted surgery and it allowed for the standardization in determining the degree of correction. Nevertheless, the formula’s versatility allows for its application with any other alignment principle that results in a specific degree of correction.

In conclusion, the overall results underscore the reliability of the proposed lateralization formula and the radiographic measurement process. The high concordance rate within the safety cone for *LAT* measurements validates the formula clinical applicability, suggesting its potential as a valuable tool in preoperative planning for TKA.

While this study demonstrates strong radiographic validation of the formula, future research will need to focus on clinical validation in the operative setting. The clinical validation will provide critical data on the formula’s real-world accuracy and its impact on achieving the intended surgical outcomes.

## Conclusion

The application of the trigonometric method was found to be reproducible and effective; its use can enable accurate planning of extramedullary tibial guidance even in the absence of dedicated software when performing personalized aligned TKA. This study’s findings support the use of the proposed formula as a dependable method for achieving desired surgical outcomes in TKA.

## Data Availability

No datasets were generated or analysed during the current study.
